# Professor David Y. Graham A Brilliant Scientist and a Compassionate Mentor

**Published:** 2018-05

**Authors:** Marjan Mohammadi

**Affiliations:** HPGC Research Group, Medical Biotechnology Department, Pasteur Institute of Iran, Tehran, Iran E-mail: marjan.mohammadi@pasteur.ac.ir


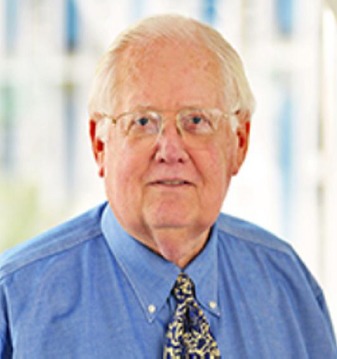


There is an enormous number of astounding scientists, who have made their mark in science. Very few of them also serve as a true mentor, ready to provide a helping hand to young researchers, especially those in developing countries, who must constantly struggle to remain optimistic. Professor David Y. Graham is one of these rare characters.

He received his undergraduate degree from the University of Notre Dame in South Bend, Indiana, his M.D. degree with honor from Baylor College of Medicine in 1966, and is board-certified in Medicine and Gastroenterology. He is currently a staff physician at the Michael E. DeBakey VA Medical Center and a professor in the Departments of Medicine and Molecular Virology and Microbiology at Baylor College of Medicine, in Houston, Texas. His first research paper was published in 1967. Since then, his research interests have varied widely, but have generally been focused on the acid-peptic diseases and the role of infectious agents in gastrointestinal disorders. He has also been interested in the exocrine pancreatic insufficiency and his first paper on this subject (as a single-authored paper) was published in the New England Journal of Medicine in 1977[1]. His latest paper on this topic was published in 2016[2]. His interest in the possible infectious etiology of inflammatory bowel disease took him into the area of viral gastroenteritis, where he, working with Dr. Mary K. Estes, made major advances in both rotavirus and norovirus gastroenteritis, including the recent report of finally being able to cultivate the previously uncultivable noroviruses *in vitro*[3]. He has continued his work on the infectious cause of Crohn’s disease and is currently the lead investigator on a multicenter trial of antimicrobials to treat *Mycobacterium paratuberculosis* in Crohn’s patients.

However, he is probably best known for his efforts related to *Helicobacter pylori*, as the cause of peptic ulcers and gastric cancer. He was among the first investigators who approached the question of whether this organism was clinically important and has published on basically all aspects of the problem. One of his highly influential work was a randomized controlled trial, which was published in the *Annals of Internal Medicine* in 1992[4], showing it was possible to cure peptic ulcer disease with antibiotics (against *Helicobacter pylori*). This paper, was assigned as a “What’s Hot in Medicine” paper by *Current Contents*, and was subsequently designated as one of the “Top Ten Advances in Medical Progress” in 1992, by the *Harvard Health Letter* and also as one of the most influential papers in Gastroenterology, published in the first 80 years of the *Annals of Internal Medicine*. This breakthrough paper has received well over 1000 citations.

Professor Graham is one of ISI’s Highly Cited Researchers, in Clinical Medicine. The results of his research have been presented in more than 1000 scientific papers and 120 chapters in medical textbooks, which have been cited by over 20,000 Scopus-accredited scientific publications. His work has resulted in a number of US patents, including the development of diagnostic tests for *Helicobacter pylori* infection (the most prominent gastric ulcerogenic and carcinogenic agent), Norwalk virus infection (the most common cause of food-borne and cruise ship-associated diarrhea), and *Mycobacterium paratuberculosis* (a causative agent for ulcerative colitis and Crohn’s disease). He is also the founding editor-in-chief of the scientific journal, *Helicobacter*, which follows his own mentality of “equal opportunity for all”, as reflected by a highly diverse geographic origin of its authors.

Professor Graham was elected as a Fellow of the American College of Physicians, the American Academy of Microbiology, the Infectious Diseases Society of America, and World Innovation Foundation and as a Master of the American College of Gastroenterology. He is the recipient of many distinguished awards, including the Joseph B. Kirsner Award for Clinical Research from the American Gastroenterology Association, the Michael E. DeBakey, M.D. Award for Excellence in Research from Baylor College of Medicine, the Janssen Award for Special Achievement in Gastroenterology from the American Gastroenterology Association, and the Frank Brown Berry Prize in Federal Medicine.

In 2008, the Endoscopy Unit at the Michael E. DeBakey VA Medical Center was officially named “The David Y. Graham Gastrointestinal Endoscopy Unit”. Professor Graham has also been honored as one of the “Best Doctors in America” annually since 1997 and as one of “Castle Connolly America’s Top Doctors” since 2008 and has been listed in the “Guide to America’s Top Gastroenterologists” since 2010. He was the president of the American College of Gastroenterology in 1990 and 1991, and since 2004, an annual lecture was named after him, which is given each year at the annual meeting of the American College of Gastroenterology. He served as the chief at Digestive Disease Division, Department of Medicine, Baylor College of Medicine, from 1988 to 2007. He continues to work, lead research and advise young investigators at Baylor College of Medicine.

During his brilliant career, Professor Graham has proudly trained more than 125 foreign physician-scientists and more than 130 U.S. Gastroenterology fellows. He remains a major mentor for many of these individuals, many of whom are now leaders in their specific disciplines. What is not usually publicly known, but is considered Professor Graham’s most exquisite feature, is his constant availability to provide advice and/or assistance to unrecognized researchers from developing countries, linking them to well-known investigators in the developed world. In addition, he routinely assists foreign-trained researchers in writing their papers in English, while accepting no recognition. He is also a strong science advocate who encourages foreign researchers to explore and write about their locally prevalent problems and submit to top journals, and thus bring their regions to the attention of interested researchers, worldwide.

In summary, Professor David Y. Graham has played a leading role in revolutionizing the understanding and care of patients with many digestive diseases. His approach has always been to link basic research findings to their clinical applications. He was one of the first in the field of Gastroenterology to understand the need for translational research, and his career has been exemplary, in this regard. To this date (at the proud age of 76), Professor Graham rounds daily with Gastroenterology fellows and residents and actively participates in the Digestive Disease Center in the Texas Medical Center, as well as in scientific conferences, held across the world. His warm and supportive presence, in such gatherings, continues to motivate his devoted mentees, from all nationalities and keep them vastly inspired.
